# Supplementary material for: Causal Relationship between Aging and Anorexia Nervosa: A White-Matter-Microstructure-Mediated Mendelian Randomization Analysis
Source: Biomedicines. 2024 Aug 16;12(8):1874. doi: 10.3390/biomedicines12081874 (PMC11351342; doi:10.3390/biomedicines12081874)
Supplement: Supplementary file 1 [file biomedicines-12-01874-s001.zip › Supplementary Figure/Figure S8/Leaveoneout plot of MR analysis for TL to ubm-b-1572.pdf]

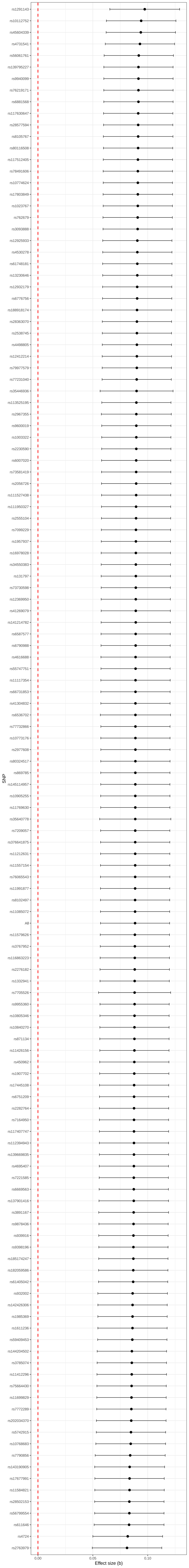

rs1291143
rs10112752
rs45604339
rs4731541
rs56061761
rs139795227
rs9940099
rs76219171
rs6881568
rs117630647
rs28577594
rs8105767
rs80116508
rs117512405
rs78491606
rs10774624
rs17803849
rs1023767
rs762679
rs3093888
rs12925933
rs4530278
rs61748181
rs13230646
rs12932179
rs6776756
rs188918174
rs28363070
rs2538745
rs4498805
rs12412214
rs79977579
rs77231040
rs35446936
rs113525195
rs2967355
rs9600019
rs1003322
rs2230590
rs6007020
rs73581419
rs2056726
rs111527438
rs111950327
rs2555104
rs7099229
rs1957937
rs16978028
rs34550383
rs131797
rs73730598
rs12369950
rs41269079
rs141214782
rs6587577
rs6790988
rs4616688
rs55747751
rs11117354
rs66731853
rs41304832
rs6536702
rs77732866
rs10773176
rs2977608
rs80324517
rs869785
rs145114957
rs10905255
rs11769630
rs35640778
rs7209057
rs376641875
rs11212631
rs11557154
rs76065543
rs11991877
rs8102497
rs11085072
A8
rs11579626
rs3767952
rs116863223
rs2276182
rs1332941
rs7705526
rs9955360
rs10805346
rs10840270
rs871134
rs11426156
rs450962
rs1907702
rs17445108
rs6751209
rs2282764
rs7164950
rs117407747
rs112394943
rs139669835
rs4695407
rs7221585
rs6669563
rs137901416
rs3891167
rs9878436
rs939916
rs9398196
rs185174247
rs182059586
rs61405042
rs932002
rs142426306
rs1985369
rs1611236
rs59409453
rs144204502
rs3785074
rs11412296
rs75664430
rs11699829
rs7772289
rs202034370
rs5742915
rs10768683
rs7790856
rs143190905
rs17677991
rs11584821
rs28502153
rs56799554
rs611646
rs4724
rs2763979
SNP
0.00
0.05
0.10
Effect size (b)
